# Livestock production: recent trends, future prospects

**DOI:** 10.1098/rstb.2010.0134

**Published:** 2010-09-27

**Authors:** Philip K. Thornton

**Affiliations:** CGIAR/ESSP Program on Climate Change, Agriculture and Food Security (CCAFS), International Livestock Research Institute (ILRI), PO Box 30709, Nairobi 00100, Kenya

**Keywords:** supply, demand, scenario, development, poverty, sustainability

## Abstract

The livestock sector globally is highly dynamic. In developing countries, it is evolving in response to rapidly increasing demand for livestock products. In developed countries, demand for livestock products is stagnating, while many production systems are increasing their efficiency and environmental sustainability. Historical changes in the demand for livestock products have been largely driven by human population growth, income growth and urbanization and the production response in different livestock systems has been associated with science and technology as well as increases in animal numbers. In the future, production will increasingly be affected by competition for natural resources, particularly land and water, competition between food and feed and by the need to operate in a carbon-constrained economy. Developments in breeding, nutrition and animal health will continue to contribute to increasing potential production and further efficiency and genetic gains. Livestock production is likely to be increasingly affected by carbon constraints and environmental and animal welfare legislation. Demand for livestock products in the future could be heavily moderated by socio-economic factors such as human health concerns and changing socio-cultural values. There is considerable uncertainty as to how these factors will play out in different regions of the world in the coming decades.

## 1. Introduction

Livestock systems occupy about 30 per cent of the planet's ice-free terrestrial surface area (Steinfeld *et al*. 2006) and are a significant global asset with a value of at least $1.4 trillion. The livestock sector is increasingly organized in long market chains that employ at least 1.3 billion people globally and directly support the livelihoods of 600 million poor smallholder farmers in the developing world (Thornton *et al*. 2006). Keeping livestock is an important risk reduction strategy for vulnerable communities, and livestock are important providers of nutrients and traction for growing crops in smallholder systems. Livestock products contribute 17 per cent to kilocalorie consumption and 33 per cent to protein consumption globally, but there are large differences between rich and poor countries (Rosegrant *et al*. 2009).

Livestock systems have both positive and negative effects on the natural resource base, public health, social equity and economic growth (World Bank 2009). Currently, livestock is one of the fastest growing agricultural subsectors in developing countries. Its share of agricultural GDP is already 33 per cent and is quickly increasing. This growth is driven by the rapidly increasing demand for livestock products, this demand being driven by population growth, urbanization and increasing incomes in developing countries (Delgado 2005).

The global livestock sector is characterized by a dichotomy between developing and developed countries. Total meat production in the developing world tripled between 1980 and 2002, from 45 to 134 million tons (World Bank 2009). Much of this growth was concentrated in countries that experienced rapid economic growth, particularly in East Asia, and revolved around poultry and pigs. In developed countries, on the other hand, production and consumption of livestock products are now growing only slowly or stagnating, although at high levels. Even so, livestock production and merchandizing in industrialized countries account for 53 per cent of agricultural GDP (World Bank 2009). This combination of growing demand in the developing world and stagnant demand in industrialized countries represents a major opportunity for livestock keepers in developing countries, where most demand is met by local production, and this is likely to continue well into the foreseeable future. At the same time, the expansion of agricultural production needs to take place in a way that allows the less well-off to benefit from increased demand and that moderates its impact on the environment.

This paper attempts a rapid summary of the present-day state of livestock production systems globally in relation to recent trends, coupled with a brief assessment of whether these trends are likely to continue into the future. In §2, the key drivers underpinning past increases in livestock production are outlined, and the status of both intensive and extensive production systems in the developed and developing world is described. Section 3 summarizes the advances in science and technology that have contributed to historical increases in livestock production, and indicates where potential remains, in relation to livestock genetics and breeding, livestock nutrition and livestock disease management. Section 4 contains sketches of a number of factors that may modify both the production and the consumption of livestock products in the future: competition for land and water, climate change, the role of socio-cultural drivers and ethical concerns. (Competition for resources and climate change are treated very briefly: other reviews address these issues comprehensively.) The section concludes with a brief discussion of three ‘wildcards’, chosen somewhat arbitrarily, that could cause considerable upheaval to future livestock production and consumption trends in the future: artificial meat, nanotechnology and deepening social concern over new technology. The paper concludes (§5) with a summary outlook on livestock production systems evolution over the coming decades and some of the key uncertainties.

## 2. Trends in livestock production and livestock systems evolution

### (a) The increasing demand for livestock products

Human population in 2050 is estimated to be 9.15 billion, with a range of 7.96–10.46 billion (UNPD 2008). Most of the increase is projected to take place in developing countries. East Asia will have shifted to negative population growth by the late 2040s (FAO 2010). In contrast, population in sub-Saharan Africa (SSA) will still be growing at 1.2 per cent per year. Rapid population growth could continue to be an important impediment to achieving improvements in food security in some countries, even when world population as a whole ceases growing sometime during the present century. Another important factor determining demand for food is urbanization. As of the end of 2008, more people now live in urban settings than in rural areas (UNFPA 2008), with urbanization rates varying from less than 30 per cent in South Asia to near 80 per cent in developed countries and Latin America. The next few decades will see unprecedented urban growth, particularly in Africa and Asia. Urbanization has considerable impact on patterns of food consumption in general and on demand for livestock products in particular: urbanization often stimulates improvements in infrastructure, including cold chains, and this allows perishable goods to be traded more widely (Delgado 2005). A third driver leading to increased demand for livestock products is income growth. Between 1950 and 2000, there was an annual global *per capita* income growth rate of 2.1 per cent (Maddison 2003). As income grows, so does expenditure on livestock products (Steinfeld *et al*. 2006). Economic growth is expected to continue into the future, typically at rates ranging from between 1.0 and 3.1 per cent (van Vuuren *et al*. 2009). Growth in industrialized countries is projected to be slower than that in developing economies (Rosegrant *et al*. 2009).

The resultant trends in meat and milk consumption figures in developing and developed countries are shown in table 1, together with estimates for 2015–2050 (FAO 2006; Steinfeld *et al*. 2006). Differences in the consumption of animal products are much greater than in total food availability, particularly between regions. Food demand for livestock products will nearly double in sub-Saharan Africa and South Asia, from some 200 kcal per person per day in 2000 to around 400 kcal per person per day in 2050. On the other hand, in most OECD countries that already have high calorie intakes of animal products (1000 kcal per person per day or more), consumption levels will barely change, while levels in South America and countries of the Former Soviet Union will increase to OECD levels (Van Vuuren *et al*. 2009).

**Table 1.** Past and projected trends in consumption of meat and milk in developing and developed countries. Data for 1980–2015 adapted from Steinfeld *et al*. (2006) and for 2030–2050 from FAO (2006). Projections are shown in italic font.

		annual *per capita* consumption		total consumption	
		meat (kg)	milk (kg)	meat (Mt)	milk (Mt)
developing	1980	14	34	47	114
	1990	18	38	73	152
	2002	28	44	137	222
	*2015*	*32*	*55*	*184*	*323*
	*2030*	*38*	*67*	*252*	*452*
	*2050*	*44*	*78*	*326*	*585*
developed	1980	73	195	86	228
	1990	80	200	100	251
	2002	78	202	102	265
	*2015*	*83*	*203*	*112*	*273*
	*2030*	*89*	*209*	*121*	*284*
	*2050*	*94*	*216*	*126*	*295*

The agricultural production sector is catering increasingly to globalized diets. Retailing through supermarkets is growing at 20 per cent per annum in countries such as China, India and Vietnam, and this will continue over the next few decades as urban consumers demand more processed foods, thus increasing the role of agribusiness (Rosegrant *et al*. 2009).

### (b) The production response

**Figure 1.**
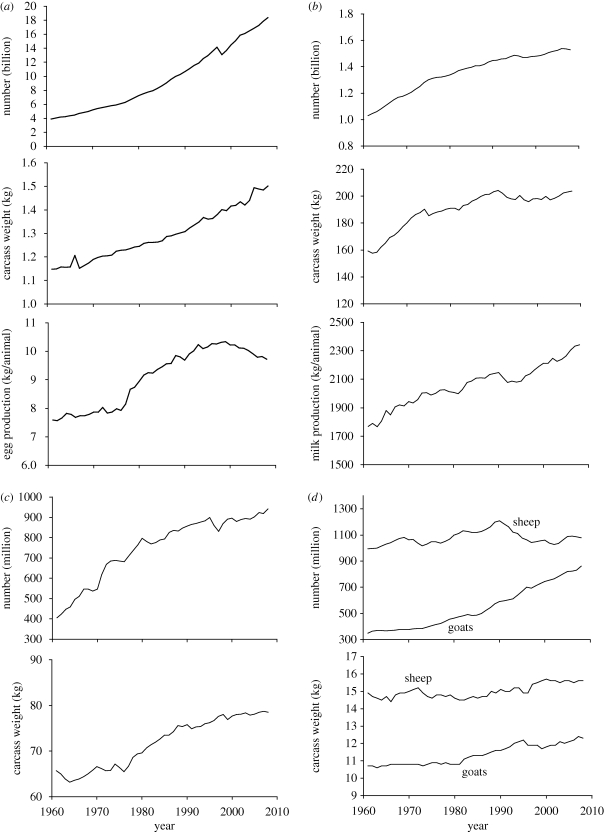

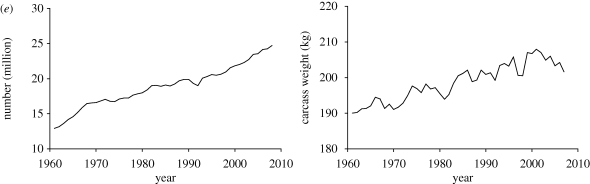
(*a*) Number of chickens, carcass weight and egg production per animal from 1961 to 2008, global. (*b*) Number of bovines (cattle and buffaloes), carcass weight and cattle milk production per animal from 1961 to 2008, global. (*c*) Number of pigs and carcass weight from 1961 to 2008, global. (*d*) Number of sheep, goats and carcass weights from 1961 to 2008, global. (*e*) Number of camels and carcass weight from 1961 to 2008, global. Data from FAO (2010).

Global livestock production has increased substantially since the 1960s. Beef production has more than doubled, while over the same time chicken meat production has increased by a factor of nearly 10, made up of increases in both number of animals and productivity (figure 1). Carcass weights increased by about 30 per cent for both chicken and beef cattle from the early 1960s to the mid-2000s, and by about 20 per cent for pigs (FAO 2010). Carcass weight increases per head for camels and sheep are much less, about 5 per cent only over this time period. Increases in milk production per animal have amounted to about 30 per cent for cows' milk, about the same as for increases in egg production per chicken over the same time period (FAO 2010).

These changes have been accompanied by substantial shifts in the area of arable land, pastures and forest. Arable and pasture lands have expanded considerably since the early 1960s, although the rates of change have started to slow (Steinfeld *et al*. 2006). Over the last 20 years, large forest conversions have occurred in the Amazon Basin, Southeast Asia and Central and West Africa, while forest area has increased owing to agricultural land abandonment in the Eurasian boreal forest and parts of Asia, North America, and Latin America and the Caribbean (LAC) (GEO4 2007). Considerable expansion of crop land planted to soybean (as a protein source in animal feed) has occurred in Latin America over the last 30 years. Developing countries' share of global use of cereals for animal feed nearly doubled (to 36%) from the early 1908s to the late 1990s (Delgado 2005). Some cropland has been converted to other uses, including urban development around many major cities. Land-use intensity has increased in some places: cereal yields have trebled in East Asia over this time, while yields have increased not at all in sub-Saharan Africa, for example. Land-use change is complex and driven by a range of drivers that are regionally specific, although it is possible to see some strong historical associations between land abundance, application of science and technology and land-use change in some regions (Rosegrant *et al*. 2009). In Latin America, for instance, land abundance has slowed the introduction of new technologies that can raise productivity.

Historically, production response has been characterized by systems' as well as regional differences. Confined livestock production systems in industrialized countries are the source of much of the world's poultry and pig meat production, and such systems are being established in developing countries, particularly in Asia, to meet increasing demand. Bruinsma (2003) estimates that at least 75 per cent of total production growth to 2030 will be in confined systems, but there will be much less growth of these systems in Africa.

While crop production growth will come mostly from yield increases rather than from area expansion, the increases in livestock production will come about more as a result of expansion in livestock numbers in developing countries, particularly ruminants. In the intensive mixed systems, food-feed crops are vital ruminant livestock feed resources. The prices of food-feed crops are likely to increase at faster rates than the prices of livestock products (Rosegrant *et al*. 2009). Changes in stover production will vary widely from region to region out to 2030 (Herrero *et al*. 2009). Large increases may occur in Africa mostly as a result of productivity increases in maize, sorghum and millet. Yet stover production may stagnate in areas such as the ruminant-dense mixed systems of South Asia, and stover will need to be replaced by other feeds in the diet to avoid significant feed deficits. The production of alternative feeds for ruminants in the more intensive mixed systems, however, may be constrained by both land and water availability, particularly in the irrigated systems (Herrero *et al*. 2009).

**Figure 2.**
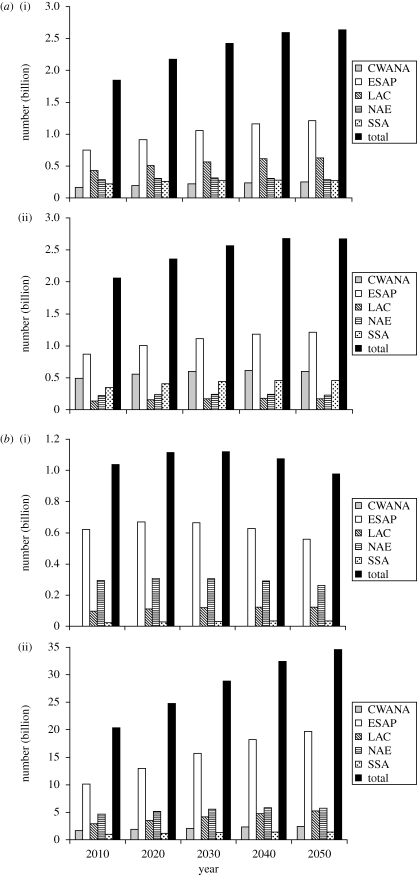
(*a*) Projected number of (i) bovines and (ii) sheep and goats to 2050 in the ‘reference world’. (*b*) Projected number of (i) pigs and (ii) poultry to 2050 in the ‘reference world’. CWANA, Central and West Asia and North Africa; ESAP, East and South Asia and the Pacific; LAC, Latin America and the Caribbean; NAE, North America and Europe; SSA, sub-Saharan Africa. Data from Rosegrant *et al*. (2009).

Meeting the substantial increases in demand for food will have profound implications for livestock production systems over the coming decades. In developed countries, carcass weight growth will contribute an increasing share of livestock production growth as expansion of numbers is expected to slow; numbers may contract in some regions. Globally, however, between 2000 and 2050, the global cattle population may increase from 1.5 billion to 2.6 billion, and the global goat and sheep population from 1.7 billion to 2.7 billion (figure 2; Rosegrant *et al*. 2009). Ruminant grazing intensity in the rangelands is projected to increase, resulting in considerable intensification of livestock production in the humid and subhumid grazing systems of the world, particularly in LAC.

The prices of meats, milk and cereals are likely to increase in the coming decades, dramatically reversing past trends. Rapid growth in meat and milk demand may increase prices for maize and other coarse grains and meals. Bioenergy demand is projected to compete with land and water resources, and this will exacerbate competition for land from increasing demands for feed resources. Growing scarcities of water and land will require substantially increased resource use efficiencies in livestock production to avoid adverse impacts on food security and human wellbeing goals. Higher prices can benefit surplus agricultural producers, but can reduce access to food by a larger number of poor consumers, including farmers who do not produce a net surplus for the market. As a result, progress in reducing malnutrition is projected to be slow (Rosegrant *et al*. 2009). Livestock system evolution in the coming decades is inevitably going to involve trade-offs between food security, poverty, equity, environmental sustainability and economic development.

## 3. Livestock science and technology as a driver of change

### (a) Breeding and genetics

Historically, domestication and the use of conventional livestock breeding techniques have been largely responsible for the increases in yield of livestock products that have been observed over recent decades (Leakey *et al*. 2009). At the same time, considerable changes in the composition of livestock products have occurred. If past changes in demand for livestock products have been met by a combination of conventional techniques, such as breed substitution, cross-breeding and within-breed selection, future changes are likely to be met increasingly from new techniques.

Of the conventional techniques, selection among breeds or crosses is a one-off process, in which the most appropriate breed or breed cross can be chosen, but further improvement can be made only by selection within the population (Simm *et al*. 2004). Cross-breeding, widespread in commercial production, exploits the complementarity of different breeds or strains and makes use of heterosis or hybrid vigour (Simm 1998). Selection within breeds of farm livestock produces genetic changes typically in the range 1–3% per year, in relation to the mean of the single or multiple traits that are of interest (Smith 1984). Such rates of change have been achieved in practice over the last few decades in poultry and pig breeding schemes in several countries and in dairy cattle breeding programmes in countries such as the USA, Canada and New Zealand (Simm 1998), mostly because of the activities of breeding companies. Rates of genetic change achieved in national beef cattle and sheep populations are often substantially lower than what is theoretically possible. Ruminant breeding in most countries is often highly dispersed, and sector-wide improvement is challenging.

Rates of genetic change have increased in recent decades in most species in developed countries for several reasons, including more efficient statistical methods for estimating the genetic merit of animals, the wider use of technologies such as artificial insemination and more focused selection on objective traits such as milk yield (Simm *et al*. 2004). The greatest gains have been made in poultry and pigs, with smaller gains in dairy cattle, particularly in developed countries and in the more industrialized production systems of some developing countries. Some of this has been achieved through the widespread use of breed substitution, which tends to lead to the predominance of a few highly specialized breeds, within which the genetic selection goals may be narrowly focused.

While most of the gains have occurred in developed countries, there are considerable opportunities to increase productivity in developing countries. Within-breed selection has not been practised all that widely, in part because of the lack of the appropriate infrastructure needed (such as performance recording and genetic evaluation schemes). Breed substitution or crossing can result in rapid improvements in productivity, but new breeds and crosses need to be appropriate for the environment and to fit within production systems that may be characterized by limited resources and other constraints. High-performing temperate breeds of dairy cow may not be appropriate for some developing-country situations: for example, heat stress and energy deficits make the use of Friesians in smallholdings on the Kenyan coast unsustainable, partly because of low cow replacement rates (King *et al*. 2006*a*). There is much more potential in the use of crosses of European breeds with local Zebus that are well-adapted to local conditions.

In the future, many developed countries will see a continuing trend in which livestock breeding focuses on other attributes in addition to production and productivity, such as product quality, increasing animal welfare, disease resistance and reducing environmental impact. The tools of molecular genetics are likely to have considerable impact in the future. For example, DNA-based tests for genes or markers affecting traits that are difficult to measure currently, such as meat quality and disease resistance, will be particularly useful (Leakey *et al*. 2009). Another example is transgenic livestock for food production; these are technically feasible, although the technologies associated with livestock are at an earlier stage of development than the equivalent technologies in plants. In combination with new dissemination methods such as cloning, such techniques could dramatically change livestock production. Complete genome maps for poultry and cattle now exist, and these open up the way to possible advances in evolutionary biology, animal breeding and animal models for human diseases (Lewin 2009). Genomic selection should be able to at least double the rate of genetic gain in the dairy industry (Hayes *et al*. 2009), as it enables selection decisions to be based on genomic breeding values, which can ultimately be calculated from genetic marker information alone, rather than from pedigree and phenotypic information. Genomic selection is not without its challenges, but it is likely to revolutionize animal breeding.

As the tools and techniques of breeding are changing, so are the objectives of many breeding programmes. Although there is little evidence of direct genetic limits to selection for yield, if selection is too narrowly focused there may be undesirable associated responses (Simm *et al*. 2004); for example, in dairy cattle, where along with genetic gain in some production traits, there is now considerable evidence of undesirable genetic changes in fertility, disease incidence and overall stress sensitivity, despite improved nutrition and general management (Hare *et al*. 2006). Trade-offs are likely to become increasingly important, between breeding for increased efficiency of resource use, knock-on impacts on fertility and other traits and environmental impacts such as methane production. Whole-system and life-cycle analyses (‘cradle-to-grave’ analyses that assess the full range of relevant costs and benefits) will become increasingly important in disentangling these complexities.

New tools of molecular genetics may have far-reaching impacts on livestock and livestock production in the coming decades. But ultimately, whether the tools used are novel or traditional, all depend on preserving access to animal genetic resources. In developing countries, if livestock are to continue to contribute to improving livelihoods and meeting market demands, the preservation of farm animal genetic resources will be critical in helping livestock adapt to climate change and the changes that may occur in these systems, such as shifts in disease prevalence and severity. In developed countries, the narrowing animal genetic resource base in many of the intensive livestock production systems demonstrates a need to maintain as broad a range of genetic resources as possible, to provide genetic insurance against future challenges and shocks. Institutional and policy frameworks that encourage the sustainable use of traditional breeds and *in situ* conservation need to be implemented, and more understanding is needed of the match between livestock populations, breeds and genes with the physical, biological and economic landscape (FAO 2007).

### (b) Nutrition

The nutritional needs of farm animals with respect to energy, protein, minerals and vitamins have long been known, and these have been refined in recent decades. Various requirement determination systems exist in different countries for ruminants and non-ruminants, which were originally designed to assess the nutritional and productive consequences of different feeds for the animal once intake was known. However, a considerable body of work exists associated with the dynamics of digestion, and feed intake and animal performance can now be predicted in many livestock species with high accuracy.

A large agenda of work still remains concerning the robust prediction of animal growth, body composition, feed requirements, the outputs of waste products from the animal and production costs. Such work could go a long way to help improve the efficiency of livestock production and meeting the expectations of consumers and the demands of regulatory authorities. Advances in genomics, transcriptomics, proteomics and metabolomics will continue to contribute to the field of animal nutrition and predictions relating to growth and development (Dumas *et al*. 2008). Better understanding of the processes involved in animal nutrition could also contribute to improved management of some of the trade-offs that operate at high levels of animal performance, such as those associated with lower reproductive performance (Butler 2000).

While understanding of the science of animal nutrition continues to expand and develop, most of the world's livestock, particularly ruminants in pastoral and extensive mixed systems in many developing countries, suffer from permanent or seasonal nutritional stress (Bruinsma 2003). Poor nutrition is one of the major production constraints in smallholder systems, particularly in Africa. Much research has been carried out to improve the quality and availability of feed resources, including work on sown forages, forage conservation, the use of multi-purpose trees, fibrous crop residues and strategic supplementation. There are also prospects for using novel feeds from various sources to provide alternative sources of protein and energy, such as plantation crops and various industrial (including ethanol) by-products. The potential of such feeds is largely unknown. Given the prevalence of mixed crop–livestock systems in many parts of the world, closer integration of crops and livestock in such systems can give rise to increased productivity and increased soil fertility (McIntire *et al*. 1992). In such systems, smallholders use crops for multiple purposes (food and feed, for example), and crop breeding programmes are now well established that are targeting stover quality as well as grain yield in crops such as maize, sorghum, millet and groundnut.

Considerable work is under way to address some of the issues associated with various antinutritional factors. These include methods to reduce the tannin content of tree and shrub material, the addition of essential oils that may be beneficial in ruminant nutrition and the use of other additives such as enzymes that can lead to beneficial effects on livestock performance. Enzymes are widely added to feeds for pigs and poultry, and these have contributed (with breeding) to the substantial gains in feed conversion efficiency that have been achieved.

What are the prospects for the future? For the mixed crop–livestock smallholder systems in developing countries, there may be places where these will intensify using the inputs and tools of high-input systems in the developed world. In the places where intensification of this nature will not be possible, there are many ways in which nutritional constraints could be addressed, based on what is locally acceptable and available. One area of high priority for additional exploration, which could potentially have broad implications for tropical ruminant nutrition, is microbial genomics of the rumen, building on current research into the breaking down of lignocellulose for biofuels (NRC 2009).

Addressing the nutritional constraints faced by pastoralists in extensive rangeland systems in the developing world is extremely difficult. While there is potential to improve livestock productivity in semi-arid and arid areas, probably the most feasible solutions require integrated application of what is already known, rather than new technology. This could involve dissemination of information from early warning systems and drought prediction, for example, so that herders can better manage the complex interactions between herd size, feed availability and rainfall (NRC 2009).

For the developed world, various drivers will shape the future of livestock nutrition. First, there is the continuing search for increased efficiency in livestock production. Margins for livestock farmers are likely to remain volatile and may be affected heavily by changes in energy prices, and increased feed conversion efficiency is one way to try to keep livestock production profitable. Public health issues will become increasingly important, such as concerns associated with the use of antibiotics in animal production, including microbiological hazards and residues in food (Vallat *et al*. 2005). The World Health Organization recommended that all subtherapeutic medical antibiotic use be stopped in livestock production in 1997, and proposed strict regulation and the phasing-out of other subtherapeutic treatments such as growth promotants; but appropriate surveillance and control programmes do not exist in many countries (Leakey *et al*. 2009). All antibiotics as growth promoters were banned in the European Union (EU) in 2006, but not all countries have made the same choice as the EU. Similarly, certain hormones can increase feed conversion efficiencies, particularly in cattle and pigs, and these are used in many parts of the world. The EU has also banned the use of hormones in livestock production. The globalization of the food supply chain will continue to raise consumer concerns for food safety and quality.

Another key driver that will affect livestock nutrition is the need (or in countries such as the UK, the legal obligation) to mitigate greenhouse gas emissions. Improved feeding practices (such as increased amounts of concentrates or improved pasture quality) can reduce methane emissions per kilogram of feed intake or per kilogram of product, although the magnitude of the latter reduction decreases as production increases. Many specific agents and dietary additives have been proposed to reduce methane emissions, including certain antibiotics, compounds that inhibit methanogenic bacteria, probiotics such as yeast culture and propionate precursors such as fumarate or malate that can reduce methane formation (Smith *et al*. 2007). Whether these various agents and additives are viable for practical use or not, and what their ultimate impacts could be on greenhouse gas mitigation, are areas that need further research.

### (c) Disease

Animal diseases generate a wide range of biophysical and socio-economic impacts that may be both direct and indirect, and may vary from localized to global (Perry & Sones 2009). The economic impacts of diseases are increasingly difficult to quantify, largely because of the complexity of the effects that they may have, but they may be enormous: the total costs of foot-and-mouth disease in the UK may have amounted to $18–25 billion between 1999 and 2002 (Bio-Era 2008).

The last few decades have seen a general reduction in the burden of livestock diseases, as a result of more effective drugs and vaccines and improvements in diagnostic technologies and services (Perry & Sones 2009). At the same time, new diseases have emerged, such as avian influenza H5N1, which have caused considerable global concern about the potential for a change in host species from poultry to man and an emerging global pandemic of human influenza.

In the developing world, there have been relatively few changes in the distribution, prevalence and impact of many epidemic and endemic diseases of livestock over the last two decades, particularly in Africa (Perry & Sones 2009), with a few exceptions such as the global eradication of rinderpest. Over this time, there has also been a general decline in the quality of veterinary services. A difficulty in assessing the changing disease status in much of the developing world is the lack of data, a critical area where progress needs to be made if disease diagnostics, monitoring and impact assessment are to be made effective and sustainable. Globally, the direct impacts of livestock diseases are decreasing, but the total impacts may actually be increasing, because in a globalized and highly interconnected world, the effects of disease extend far beyond animal sickness and mortality (Perry & Sones 2009).

For the future, the infectious disease threat will remain diverse and dynamic, and combating the emergence of completely unexpected diseases will require detection systems that are flexible and adaptable in the face of change (King *et al*. 2006*b*). Travel, migration and trade will all continue to promote the spread of infections into new populations. Trade in exotic species and in bush meat are likely to be increasing causes of concern, along with large-scale industrial production systems, in which conditions may be highly suitable for enabling disease transmission between animals and over large distances (Otte *et al*. 2007).

Over the long term, future disease trends could be heavily modified by climate change. For some vector-borne diseases such as malaria, trypanosomiasis and bluetongue, climate change may shift the geographical areas where the climate is suitable for the vector, but these shifts are not generally anticipated to be major over the next 20 years: other factors may have much more impact on shifting vector distributions in the short term (Woolhouse 2006). Even so, Van Dijk *et al*. (2010) have found evidence that climate change, especially elevated temperature, has already changed the overall abundance, seasonality and spatial spread of endemic helminths in the UK. This has obvious implications for policy-makers and the sheep and cattle industries, and raises the need for improved diagnosis and early detection of livestock parasitic disease, along with greatly increased awareness and preparedness to deal with disease patterns that are manifestly changing.

Climate change may have impacts not only on the distribution of disease vectors. Some diseases are associated with water, which may be exacerbated by flooding and complicated by inadequate water access. Droughts may force people and their livestock to move, potentially exposing them to environments with health risks to which they have not previously been exposed. While the direct impacts of climate change on livestock disease over the next two to three decades may be relatively muted (King *et al*. 2006*b*), there are considerable gaps in knowledge concerning many existing diseases of livestock and their relation to environmental factors, including climate.

Future disease trends are likely to be heavily modified by disease surveillance and control technologies. Potentially effective control measures already exist for many infectious diseases, and whether these are implemented appropriately could have considerable impacts on future disease trends. Recent years have seen considerable advances in the technology that can be brought to bear against disease, including DNA fingerprinting for surveillance, polymerase chain reaction tests for diagnostics and understanding resistance, genome sequencing and antiviral drugs (Perry & Sones 2009). There are also options associated with the manipulation of animal genetic resources, such as cross-breeding to introduce genes into breeds that are otherwise well-adapted to the required purposes, and the selection via molecular genetic markers of individuals with high levels of disease resistance or tolerance.

The future infectious disease situation is going to be different from today's (Woolhouse 2006), and will reflect many changes, including changes in mean climate and climate variability, demographic change and different technologies for combating infectious diseases. The nature of most, if not all, of these changes is uncertain, however.

## 4. Possible modifiers of future livestock production and consumption trends

### (a) Competition for resources

#### (i) Land

Recent assessments expect little increase in pasture land (Bruinsma 2003; MA 2005). Some intensification in production is likely to occur in the humid–subhumid zones on the most suitable land, where this is feasible, through the use of improved pastures and effective management. In the more arid–semiarid areas, livestock are a key mechanism for managing risk, but population increases are fragmenting rangelands in many places, making it increasingly difficult for pastoralists to gain access to the feed and water resources that they have traditionally been able to access. In the future, grazing systems will increasingly provide ecosystem goods and services that are traded, but how future livestock production from these systems may be affected is not clear. The mixed crop–livestock systems will continue to be critical to future food security, as two-thirds of the global population live in these systems. Some of the higher potential mixed systems in Africa and Asia are already facing resource pressures, but there are various responses possible, including efficiency gains and intensification options (Herrero *et al*. 2010). Increasing competition for land in the future will also come from biofuels, driven by continued concerns about climate change, energy security and alternative income sources for agricultural households. Future scenarios of bioenergy use vary widely (Van Vuuren *et al*. 2009), and there are large evidence gaps concerning the likely trade-offs between food, feed and fuel in production systems in both developed and developing countries, particularly related to second-generation bioenergy technology.

#### (ii) Water

Globally, freshwater resources are relatively scarce, amounting to only 2.5 per cent of all water resources (MA 2005). Groundwater also plays an important role in water supply: between 1.5 and 3 billion people depend on groundwater for drinking, and in some regions water tables are declining unremittingly (Rodell *et al*. 2009). By 2025, 64 per cent of the world's population will live in water-stressed basins, compared with 38 per cent today (Rosegrant *et al*. 2002). Increasing livestock numbers in the future will clearly add to the demand for water, particularly in the production of livestock feed: one cubic metre of water can produce anything from about 0.5 kg of dry animal feed in North American grasslands to about 5 kg of feed in some tropical systems (Peden *et al*. 2007). Several entry points for improving global livestock water productivity exist, such as increased use of crop residues and by-products, managing the spatial and temporal distribution of feed resources so as to better match availability with demand and managing systems so as to conserve water resources (Peden *et al*. 2007). More research is needed related to livestock–water interactions and integrated site-specific interventions, to ensure that livestock production in the future contributes to sustainable and productive use of water resources (Peden *et al*. 2007).

### (b) Climate change

Climate change may have substantial effects on the global livestock sector. Livestock production systems will be affected in various ways (table 2 and see Thornton *et al*. (2009) for a review), and changes in productivity are inevitable. Increasing climate variability will undoubtedly increase livestock production risks as well as reduce the ability of farmers to manage these risks. At the same time, livestock food chains are major contributors to greenhouse gas emissions, accounting for perhaps 18 per cent of total anthropogenic emissions (Steinfeld *et al*. 2006). Offering relatively fewer cost-effective options than other sectors such as energy, transport and buildings, agriculture has not yet been a major player in the reduction of greenhouse gas emissions. This will change in the future (UNFCCC 2008), although guidance will be needed from rigorous analysis; for example, livestock consumption patterns in one country are often associated with land-use changes in other countries, and these have to be included in national greenhouse gas accounting exercises (Audsley *et al*. 2009).

**Table 2.** Direct and indirect impacts of climate change on livestock production systems (adapted from Thornton & Gerber 2010).

grazing systems	non-grazing systems
direct impacts	
extreme weather events	water availability
drought and floods	extreme weather events
productivity losses (physiological stress) owing to temperature increase	
water availability	
indirect impacts	
agro-ecological changes:	increased resource price, e.g. feed and energy
fodder quality and quality	disease epidemics
host–pathogen interactions	increased cost of animal housing, e.g. cooling systems
disease epidemics	

Climate change will have severely deleterious impacts in many parts of the tropics and subtropics, even for small increases in the average temperature. This is in contrast to many parts of the temperate zone; at mid- to high latitudes, agricultural productivity is likely to increase slightly for local mean temperature increases of 1–3°C (IPCC 2007). There is a burgeoning literature on adaptation options, including new ways of using weather information to assist rural communities in managing the risks associated with rainfall variability and the design and piloting of livestock insurance schemes that are weather-indexed (Mude 2009). Many factors determine whether specific adaptation options are viable in particular locations. More extensive adaptation than is currently occurring is needed to reduce vulnerability to future climate change, and adaptation has barriers, limits and costs (IPCC 2007).

Similarly, there is a burgeoning literature on mitigation in agriculture. There are several options related to livestock, including grazing management and manure management. Global agriculture could offset 5–14% (with a potential maximum of 20%) of total annual CO_2_ emissions for prices ranging from $20 to 100 per t CO_2_ eq (Smith *et al*. 2008). Of this total, the mitigation potential of various strategies for the land-based livestock systems in the tropics amounts to about 4 per cent of the global agricultural mitigation potential to 2030 (Thornton & Herrero submitted), which could still be worth of the order of $1.3 billion per year at a price of $20 per t CO_2_ eq. Several of these mitigation options also have adaptive benefits, such as growing agroforestry species that can sequester carbon, which can also provide high-quality dietary supplements for cattle. Such carbon payments could represent a relatively large amount of potential income for resource-poor livestock keepers in the tropics. In the more intensive systems, progress could be made in mitigating GHG emissions from the livestock sector via increases in the efficiency of production using available technology, for the most part, and this may involve some shifting towards monogastric species.

### (c) Socio-cultural modifiers

Social and cultural drivers of change are having profound effects on livestock systems in particular places, although it is often unclear how these drivers play out in relation to impacts on livestock and livestock systems. Livestock have multiple roles in human society. They contribute substantially and directly to food security and to human health. For poor and under-nourished people, particularly children, the addition of modest amounts of livestock products to their diets can have substantial benefits for physical and mental health (Neumann *et al*. 2003).

Livestock's contribution to livelihoods, particularly those of the poor in developing countries, is also well recognized. Livestock generate income by providing both food and non-food products that the household can sell in formal or informal markets. Non-food products such as wool, hides and skins are important sources of income in some regions: wool production in the high-altitude tropical regions of Bolivia, Peru or Nepal, for example. Hides and skins from home-slaughtered animals are rarely processed, as the returns may not justify the costs involved (Otte & Upton 2005). Livestock acquisition as a pathway out of poverty has been documented by Kristjanson *et al*. (2004) in western Kenya, for example. Livestock provide traction mainly in irrigated, densely populated areas, and allow cropping in these places. They provide nutrients in the form of manure, a key resource particularly for the mixed systems of sub-Saharan Africa. Livestock also serve as financial instruments, by providing households with an alternative for storing savings or accumulated capital, and they can be sold and transformed into cash as needed and so also provide an instrument of liquidity, consumption smoothing and insurance. For some poorer households, livestock can provide a means of income diversification to help deal with times of stress.

In addition to their food security, human health, economic and environmental roles, livestock have important social and cultural roles. In many parts of Africa, social relationships are partly defined in relation to livestock, and the size of a household's livestock holding may confer considerable social importance on it. The sharing of livestock with others is often a means to create or strengthen social relationships, through their use as dowry or bride price, as allocations to other family members and as loans (Kitalyi *et al*. 2005). Social status in livestock-based communities is often associated with leadership and access to (and authority over) natural, physical and financial resources.

Livestock may have considerable cultural value in developed countries also. Local breeds have often been the drivers of specific physical landscapes (e.g. extensive pig farming in the Mediterranean oak forests of the Iberian peninsula); as such, local breeds can be seen as critical elements of cultural networks (Gandini & Villa 2003).

Compared with the biophysical environment, the social and cultural contexts of livestock and livestock production are probably not that well understood, but these contexts are changing markedly in some places. External pressures are being brought to bear on traditional open-access grazing lands in southern Kenya, for example, such as increasing population density and increasing livestock–wildlife competition for scarce resources. At the same time, many Maasai feel that there is no option but to go along with subdivision, a process that is already well under way in many parts of the region, because they see it as the only way in which they can gain secure tenure of their land and water, even though they themselves are well aware that subdivision is likely to harm their long-term interests and wellbeing (Reid *et al*. 2008). There are thus considerable pressures on Maasai communities and societies, as many households become more connected to the cash economy, access to key grazing resources becomes increasingly problematic, and cultural and kinship networks that have supported them in the past increasingly feel the strain. Inevitably, the cultural and social roles of livestock will continue to change, and many of the resultant impacts on livelihoods and food security may not be positive.

Social and cultural changes are likewise taking place elsewhere. In European agriculture, there is already heightened emphasis on, and economic support for, the production of ecosystems goods and services, and this will undoubtedly increase in the future (Deuffic & Candau 2006). In the uplands of the UK, recent social changes have seen increasing demand for leisure provision and access to rural areas. At the same time, there are increasing pressures on the social functions and networks associated with the traditional farming systems of these areas, which have high cultural heritage value and considerable potential to supply the public goods that society is likely to demand in the future (Burton *et al*. 2005).

### (d) Ethical concerns as a driver of change

Ethical concerns may play an increasing role in affecting the production and consumption of livestock products. Recent high-profile calls to flock to the banner of global vegetarianism, backed by exaggerated claims of livestock's role in anthropogenic global greenhouse gas emissions, serve mostly to highlight the need for rigorous analysis and credible numbers that can help inform public debate about these issues: there is much work to do in this area.

But science has already had a considerable impact on some ethical issues. Research into animal behaviour has provided evidence of animals' motivations and their mental capacities, which by extension provides strong support for the notion of animal sentience (i.e. animals' capacity to sense and feel), which in turn has provided the basis for EU and UK legislation that enshrines the concept of animal sentience in law (Lawrence 2009). Recently, European government strategies are tending to move away from legislation as the major mechanism for fostering animal welfare improvements to a greater concentration on collective action on behalf of all parties with interests in animal welfare, including consumers (Lawrence 2008). There is conflicting evidence as to the potential for adding value to animal products through higher welfare standards. There are common questions regarding the robustness of consumers' preferences regarding welfare-branded, organic and local food, for example, particularly in times of considerable economic uncertainty.

While there are differences between different countries in relation to animal welfare legislation, animal welfare is an increasingly global concern. Part of this probably arises as a result of the forces of globalization and international trade, but in many developing countries the roots of animal welfare may be different and relate more to the value that livestock have to different societies: the sole or major source of livelihood (in some marginal environments in SSA, for example), the organizing principle of society and culture (the Maasai, for instance), investment and insurance vehicles and sources of food, traction and manure, for example (Kitalyi *et al*. 2005).

Improving animal welfare need not penalize business returns and indeed may increase profits. For instance (and as noted above), measurements of functional traits indicate that focusing on breeding dairy cows for milk yield alone is unfavourably correlated with reductions in fertility and health traits (Lawrence *et al*. 2004). The most profitable bulls are those that produce daughters that yield rather less milk but are healthier and longer lived: the costs of producing less milk can be more than matched by the benefits of decreased health costs and a lower herd replacement rate. Identifying situations where animal welfare can be increased along with profits, and quantifying these trade-offs, requires integrated assessment frameworks that can handle the various and often complex inter-relationships between animal welfare, management and performance (Lawrence & Stott 2009).

### (e) Wildcard drivers of change

There is considerable uncertainty related to technological development and to social and cultural change. This section briefly outlines an arbitrary selection of wildcards, developments that could have enormous implications for the livestock sector globally, either negatively (highly disruptive) or positively (highly beneficial).

#### (i) Artificial meat (more correctly, in vitro meat)

From a technological point of view, this may not be a wildcard at all, as its development is generally held to be perfectly feasible (Cuhls 2008), and indeed research projects on it have been running for a decade already. There are likely to be some issues associated with social acceptability, although presumably meat ‘grown in vats’ could be made healthier by changing its composition and made much more hygienic than traditional meat, as it would be cultured in sterile conditions. *In vitro* meat could potentially bypass many of the public health issues that are currently associated with livestock-based meat. The development and uptake of *in vitro* meat on a large scale would unquestionably be hugely disruptive to the traditional livestock sector. It would raise critical issues regarding livestock keeping and livelihoods of the resource-poor in many developing countries, for example. On the other hand, massive reductions in livestock numbers could contribute substantially to the reduction of greenhouse gases, although the net effects would depend on the resources needed to produce *in vitro* meat. There are many issues that would need to be considered, including the effects on rangelands of substantial decreases in the number of domesticated grazing animals, and some of the environmental and socio-cultural impacts would not be positive. There could also be impacts on the amenity value of landscapes with no livestock in some places. Commercial *in vitro* meat production is not likely to happen any time soon, however: at least another decade of research is needed, and then there will still be the challenges of scale and cost to be overcome.

#### (ii) Nanotechnology

This refers to an extremely dynamic field of research and application associated with particles of 1–100 nm in size (the size range of many molecules). Some particles of this size have peculiar physical and chemical properties, and it is such peculiarities that nanotechnology seeks to exploit. Nanotechnology is a highly diverse field, and includes extensions of conventional device physics, completely new approaches based upon molecular self-assembly and the development of new materials with nanoscale dimensions. There is even speculation as to whether matter can be directly controlled at the atomic scale. Some food and nutrition products containing nanoscale additives are already commercially available, and nanotechnology is in widespread use in advanced agrichemicals and agrichemical application systems (Brunori *et al*. 2008). The next few decades may well see nanotechnology applied to various areas in animal management. Nanosized, multipurpose sensors are already being developed that can report on the physiological status of animals, and advances can be expected in drug delivery methods using nanotubes and other nanoparticles that can be precisely targeted. Nanoparticles may be able to affect nutrient uptake and induce more efficient utilization of nutrients for milk production, for example. One possible approach to animal waste management involves adding nanoparticles to manure to enhance biogas production from anaerobic digesters or to reduce odours (Scott 2006). There are, however, considerable uncertainties concerning the possible human health and environmental impacts of nanoparticles, and these risks will have to be addressed by regulation and legislation: at present, for all practical purposes, nanotechnology is unregulated (Speiser 2008). Brunori *et al*. (2008) see nanotechnology as potentially a highly disruptive driver, and the ongoing debate as to the pros and cons is currently not well informed by objective information on the risks involved: much more information is required on its long-term impacts. Nanotechnology could redefine the entire notion of agriculture and many other human activities (Cuhls 2008).

#### (iii) Deepening social concerns about specific technology

Much evidence points to a serious disconnect between science and public perceptions. Marked distrust of science is a recurring theme in polls of public perceptions of nuclear energy, genetic modification and, spectacularly, anthropogenic global warming. One of several key reasons for this distrust is a lack of credible, transparent and well-communicated risk analyses associated with many of the highly technological issues of the day. This lack was noted above in relation to nanotechnology, but it applies in many other areas as well. The tools of science will be critical for bringing about food security and wellbeing for a global population of more than nine billion people in 2050 in the face of enormous technological, climatic and social challenges. Technology is necessary for the radical redirection of global food systems that many believe is inevitable, but technology alone is not sufficient: the context has to be provided whereby technology can build knowledge, networks and capacity (Kiers *et al*. 2008). One area where there are numerous potential applications to agriculture is the use of transgenic methodology to develop new or altered strains of livestock. These applications include ‘… improved milk production and composition, increased growth rate, improved feed usage, improved carcass composition, increased disease resistance, enhanced reproductive performance, and increased prolificacy’ (Wheeler 2007, p. 204). Social concerns could seriously jeopardize even the judicious application of such new science and technology in providing enormous economic, environmental and social benefits. If this is to be avoided, technology innovation has to take fully into account the health and environmental risks to which new technology may give rise. Serious and rapid attention needs to be given to risk analysis and communications policy.

## 5. Conclusions

What is the future for livestock systems globally? Several assessments agree that increases in the demand for livestock products, driven largely by human population growth, income growth and urbanization, will continue for the next three decades at least. Globally, increases in livestock productivity in the recent past have been driven mostly by animal science and technology, and scientific and technological developments in breeding, nutrition and animal health will continue to contribute to increasing potential production and further efficiency and genetic gains. Demand for livestock products in the future, particularly in developed countries, could be heavily moderated by socio-economic factors such as human health concerns and changing socio-cultural values.

In the future, livestock production is likely to be increasingly characterized by differences between developed and developing countries, and between highly intensive production systems on the one hand and smallholder and agropastoral systems on the other. How the various driving forces will play out in different regions of the world in the coming decades is highly uncertain, however. Of the many uncertainties, two seem over-arching. First, can future demand for livestock products be met through sustainable intensification in a carbon-constrained economy? Some indications have been given above of the increasing pressures on natural resources such as water and land; the increasing demand for livestock products will give rise to considerable competition for land between food and feed production; increasing industrialization of livestock production may lead to challenging problems of pollution of air and water; the biggest impacts of climate change are going to be seen in livestock and mixed systems in developing countries where people are already highly vulnerable; the need to adapt to climate change and to mitigate greenhouse emissions will undoubtedly add to the costs of production in different places; and the projected growth in biofuels may have substantial additional impacts on competition for land and on food security.

A second over-arching uncertainty is, will future livestock production have poverty alleviation benefits? The industrialization of livestock production in many parts of the world, both developed and developing, is either complete or continuing apace. The increasing demand for livestock products continues to be a key opportunity for poverty reduction and economic growth, although the evidence of the last 10 years suggests that only a few countries have taken advantage of this opportunity effectively (Dijkman 2009). Gura (2008) documents many cases where the poor have been disadvantaged by the industrialization of livestock production in developing countries, as well as highlighting the problems and inadequacies of commercial, industrial breeding lines, once all the functions of local breeds are genuinely taken into account. The future role of smallholders in global food production and food security in the coming decades is unclear. Smallholders currently are critical to food security for the vast majority of the poor, and this role is not likely to change significantly in the future, particularly in SSA. But increasing industrialization of livestock production may mean that smallholders continue to miss out on the undoubted opportunities that exist. There is no lack of suggestions as to what is needed to promote the development of sustainable and profitable smallholder livestock production: significant and sustained innovation in national and global livestock systems (Dijkman 2009); increasing regulation to govern contracts along food commodity chains, including acceptance and guarantee of collective rights and community control (Gura 2008); and building social protection and strengthening links to urban areas (Wiggins 2009). Probably all of these things are needed, headed by massive investment, particularly in Africa (World Bank 2009).

It is thought that humankind's association with domesticated animals goes back to around 10 000 BC, a history just about as long as our association with domesticated plants. What is in store for this association during the coming century is far from clear, although it is suffering stress and upheaval on several fronts. The global livestock sector may well undergo radical change in the future, but the association is still critical to the wellbeing of millions, possibly billions, of people: in many developing countries, at this stage in history, it has no known, viable substitute.
